# Infrared Spectroscopy of Fluorenyl Cations at Cryogenic Temperatures

**DOI:** 10.1021/acs.jpclett.3c02928

**Published:** 2023-12-08

**Authors:** Kim Greis, Carla Kirschbaum, Katja Ober, Martín I. Taccone, América Y. Torres-Boy, Gerard Meijer, Kevin Pagel, Gert von Helden

**Affiliations:** † Fritz Haber Institute of the Max Planck Society, Berlin, Faradayweg 4-6, 14195 Berlin, Germany; ‡ Institute of Chemistry and Biochemistry, Freie Universität Berlin, Altensteinstraße 23A, 14195 Berlin, Germany

## Abstract

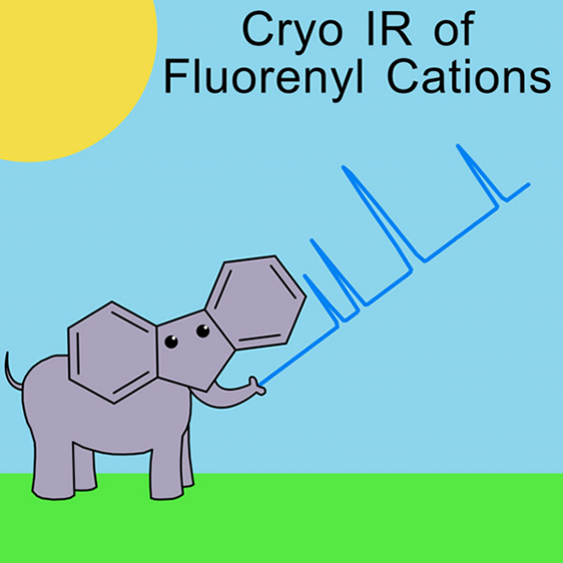

The notion of (anti)aromaticity is a successful concept in chemistry to explain the structure and stability of polycyclic hydrocarbons. Cyclopentadienyl and fluorenyl cations are among the most studied classical antiaromatic systems. In this work, fluorenyl cations are investigated by high-resolution gas-phase infrared spectroscopy in helium droplets. Bare fluorenyl cations are generated in the gas phase by electrospray ionization. After mass-to-charge selection, ions are captured in ultracold helium nanodroplets and probed by infrared spectroscopy using a widely tunable free-electron laser in the 600–1700 cm^–1^ range. The highly resolved cryogenic infrared spectra confirm, in combination with DFT computations, that all cations are present in their singlet states.

The distribution of electrons in molecules and concepts such as electron localization and delocalization are fundamental in chemistry and are used to describe the structure, stability, and reactivity of molecules. For cyclic and polycyclic organic carbon-containing molecules, the 4*n* + 2 π electron Hückel rule has been particularly successful in explaining enhanced stabilities. Contrary to aromatic 4*n* + 2 π electron systems, 4*n* π electron systems are particularly unstable and were termed “antiaromatic” by Breslow.^1^ A prime example is the cyclopentadienyl cation (Scheme 1), which should be antiaromatic in its singlet state. However, according to Baird’s rule,^2^ the situation is reversed for triplet states where 4*n* π electron systems are expected to behave aromatic.

**Scheme 1:**
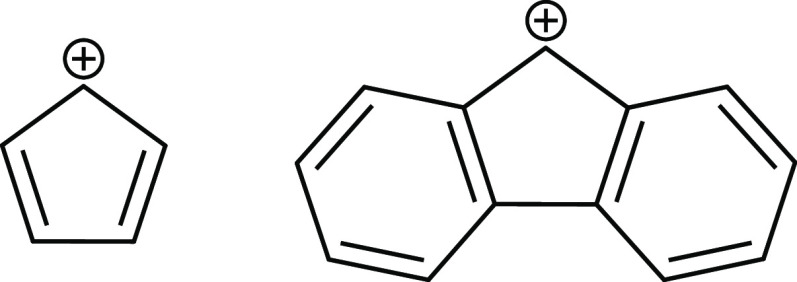
Structures of the Cyclopentadienyl Cation (C_5_H_5_^+^, Left) and the 9-Fluorenyl Cation Fl^+^ (C_13_H_9_^+^, Right)

The related and potentially antiaromatic 9-fluorenyl cation (Scheme 1) has also intrigued researchers for many decades. However, due to its high reactivity and short lifetime, it is challenging to analyze and early experiments to stabilize unsubstituted fluorenyl cations in superacidic media failed due to polymerization.^3^ However, measurements of the solvolysis rates of the hydroxylated precursors of several 9-fluorenyl cations and related species led to the conclusion that the description of the 9-fluorenyl cation as antiaromatic is misleading.^4^

Fluorenyl cations substituted at the C9 position (the top position of the five-membered ring), on the other hand, are easier to generate and characterize. It is possible to stabilize 9-fluorenyl cations with alkyl, phenyl, hydroxy, and chloro groups in superacids and analyze their structure with ^1^H and ^13^C NMR spectroscopy.^3^ Furthermore, tetrachloroaluminate crystals of a fluorenyl cation with a hydroxy group at the C9 position and methyl and mesityl substituents on the annulated benzyl rings are formed. Although the compound degrades in chlorinated NMR solvents within 1 day and as a solid under inert conditions within weeks, its NMR spectra and X-ray structure have been measured.^5^ In a recent study, a fluorenyl cation has been stabilized with diaminomethyl substituents, exhibiting a lifetime of minutes in moderately protic solvents.^6^ The reported NMR-shifts, as well as computed nucleus-independent chemical shift values, do not support antiaromaticity in the case of fluorenyl cation derivatives. While the five-membered ring itself might be described as antiaromatic, it is highly stabilized through the annulated benzene rings and potential substituents at the C9 position.

The unsubstituted 9-fluorenyl cation can be generated as a short-lived intermediate in ultrafast UV–vis spectroscopy experiments. The ion was shown to have lifetimes of picoseconds in methanol^7,8^ and microseconds in certain zeolites,^9^ limiting detailed investigations. An alternative strategy is to study the 9-fluorenyl cation in isolation, either in the gas phase or in a nonreactive environment. Upon ionization of fluorene in the gas phase using UV light, the 9-fluorenyl cation has been identified as a fragmentation product, and some broad IR absorption bands have been tentatively assigned to it.^10^ In another study, mass-to-charge selected C_13_H_9_^+^ from the ionization and fragmentation of fluorene has been deposited in a neon matrix to record its electronic absorption spectrum.^11^ Further, an infrared (IR) spectrum of the 9-fluorenyl cation has been measured by matrix-isolation spectroscopy in low-density amorphous water ice after the photolysis of diazofluorene, followed by protonation. The accompanying calculations are in very good agreement with the experimental data, and the authors conclude that the presence of the water ice matrix introduces only negligible shifts.^12^

An ideal matrix for performing spectroscopic experiments is superfluid helium. We recently introduced a technique in which mass-to-charge selected molecular ions are implanted into superfluid helium droplets that have an equilibrium temperature of 0.4 K. This allows for the spectroscopic investigation of ions at ultralow temperature, almost free of interactions with the surroundings and free of interactions with counterions. This technique has been previously applied to characterize various ions, including FH_2_CO_3_^–^,^13^ protein ions,^14^ and the reactive intermediate of the glycosylation reaction.^15,16^

Here we investigate a set of 9-fluorenyl cations using vibrational spectroscopy under ultracold conditions. The ions are generated using nanoelectrospray ionization (nESI) followed by mass spectrometry and resonant IR excitation of mass-to-charge selected ions in helium nanodroplets. The resulting spectra consist of narrow bands and allow for the assignment of the spin state and structure.

The experimental setup has been previously described in detail.^13−17^ Fluorenyl cations are generated by nESI and subsequent in-source fragmentation of precursor molecules carrying an appropriate leaving group or protonation of precursors with a carbonyl group (Figures 1 and S1). 9-Fluorenyl cations (**Fl**^**+**^; C_13_H_9_^+^) are generated by in-source fragmentation of 9-fluorenyl methacrylate, whereas 9-phenyl-9-fluorenyl cations (**PhFl**^**+**^; C_19_H_13_^+^) readily form upon ESI of 9-bromo-9-phenylfluorene. Interestingly, no parent ion signals were visible for the bromo precursors. Hence, bromide leaving groups are cleaved easily under the employed ionization conditions. 9-Hydroxy-9-fluorenyl cations (**HOFl**^**+**^; C_13_H_9_O^+^), on the other hand, readily form during ESI upon protonation of 9-fluorenone.

**Figure 1:**
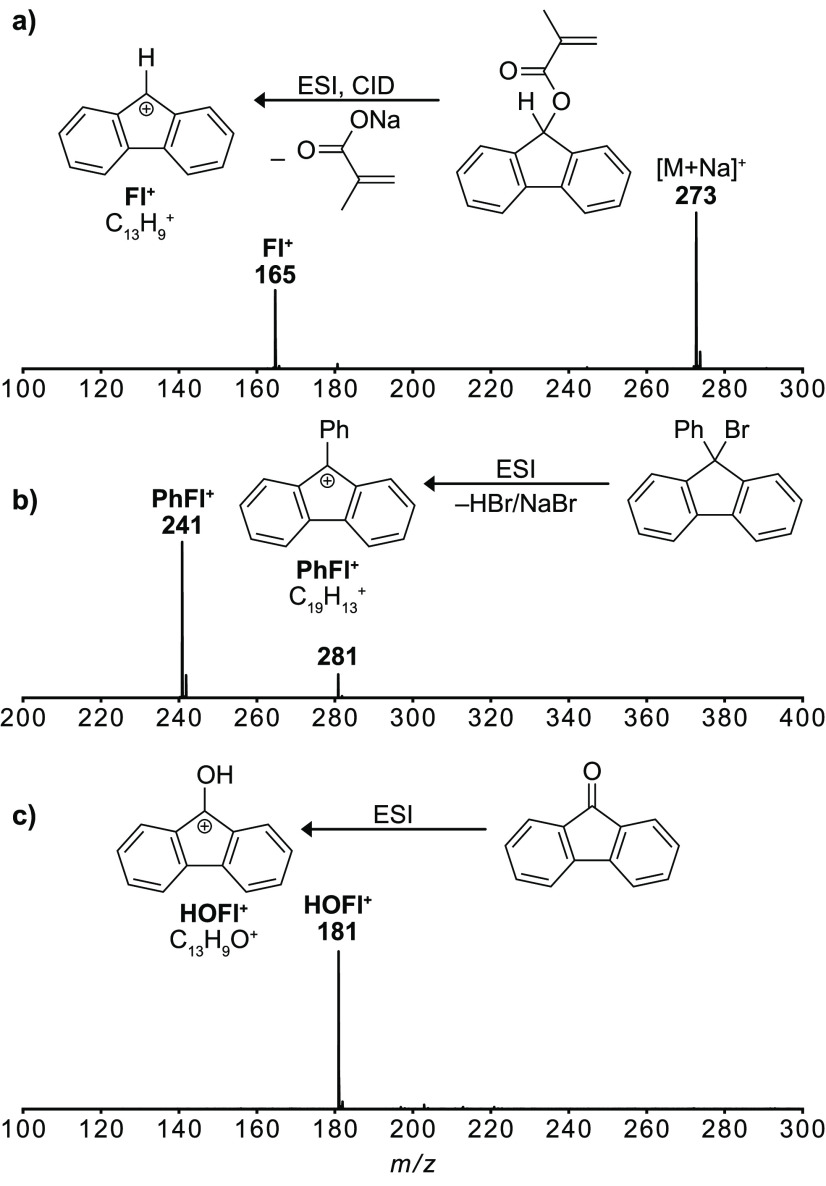
ESI-(+)-mass spectra of depicted precursors leading to fluorenyl cations: (a) 9-fluorenyl methacrylate, leading to the 9-fluorenyl cation **Fl**^**+**^ (*m*/*z* 165); (b) 9-bromo-9-phenylfluorene, leading to the 9-phenyl-9-fluorenyl cation **PhFl**^**+**^ (*m*/*z* 241); (c) 9-fluorenone, leading to the 9-hydroxyl-9-fluorenyl cation **HOFl**^**+**^ (*m*/*z* 181).

Figure 2 shows the cryogenic IR spectra of **Fl**^**+**^, **PhFl**^**+**^, and **HOFl**^**+**^. Sharp resonances can be observed with widths that are limited by the bandwidth of the Fritz Haber Institute free-electron laser (FHI-FEL, ∼0.4% of the respective wavenumber). The experimental spectrum can be compared with results from computations. Although the ions differ only in the substituent at the C9 position, the spectral signatures are significantly different. The positions of the absorption bands of **Fl**^**+**^ agree with those previously recorded using matrix isolation spectroscopy in water ice in the 900–1650 cm^–1^ region^12^ (see Table S2 and Figure S2). However, the absorption bands at 1009 and 1583 cm^–1^ were not mentioned by Costa et al., whereas the absorption band at 1117 cm^–1^ exhibits only a very weak intensity in cryogenic IR spectroscopy.

**Figure 2:**
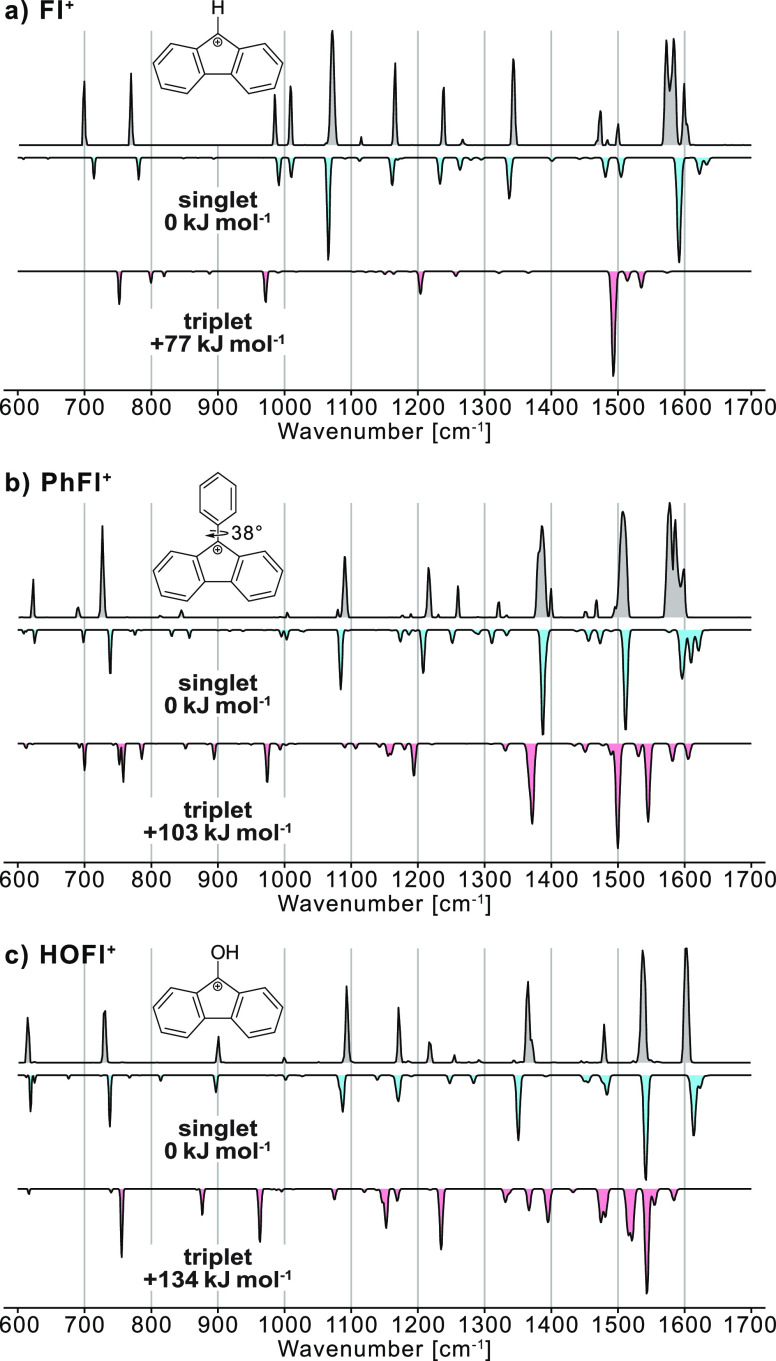
Infrared spectra of (a) the 9-fluorenyl cation **Fl**^**+**^, (b) the 9-phenylfluorenyl cation **PhFl**^**+**^, and (c) the 9-hydroxyfluorenyl cation **HOFl**^**+**^. Experimental cryogenic gas-phase infrared spectra are shown as gray traces. Computed spectra for the singlet (blue) and triplet states (red) are shown in the inverted traces and originate from harmonic frequency calculations at the CAM-B3LYP+D3/def2-TZVPP level of theory. The structure and relative energies are indicated.

The experimental IR spectra are compared with the respective computed harmonic frequencies for singlet and triplet states (Figure 2). For this endeavor, three density functionals are chosen: CAM-B3LYP, B3LYP, and PBE0 with the basis set def2-TZVPP and Grimme’s D3 dispersion correction with Becke–Johnson damping. CAM-B3LYP produced overall the best matching harmonic frequencies, whereas some harmonic frequencies match better for PBE0 or B3LYP, as shown in Figures S2–S4. For all three ions, the IR signatures of the singlet and triplet ions differ significantly, and the calculated singlet spectra agree very well with the respective experimental spectra. We can thus safely state that the ground states of **Fl**^**+**^, **PhFl**^**+**^, and **HOFl**^**+**^ are singlet electronic states. This result is in line with theory, which predicts triplet states that are significantly higher in energy than the singlet states for all three ions. The singlet–triplet gap increases with an increasing mesomeric π-donation effect of the substituent.

The **Fl**^**+**^ cation is planar and exhibits a *C*_2*v*_-symmetry. All high intensity modes of the calculated spectrum of the singlet state of **Fl**^**+**^ are in-plane bond stretching and bending modes, except for the vibrational bands at 700 and 770 cm^–1^, which correspond to out-of-plane C–H rocking modes. The band calculated at 984 cm^–1^ is a breathing mode of the two benzene rings. Higher in wavenumber, the modes at 1072 and 1153 cm^–1^ have almost exclusive C–H bend character. The weak mode at 1234 cm^–1^, the stronger mode at 1330 cm^–1^, and the mode calculated at 1468 cm^–1^ have C–H bend character with some C–C stretch and bend motion mixed in, while the signals above 1500 cm^–1^ have mainly C=C stretch character. There are two absorption bands at 1572 and 1583 cm^–1^, whereas computed harmonic spectra at different levels of theory (Figure S2) mainly predict one matching band in this region. The origin of this discrepancy is unclear. The position of experimental and calculated frequencies can be found in Table S2.

The **PhFl**^**+**^ cation, on the other hand, is *C*_2_-symmetric, as the phenyl substituent at the C9 position is, despite the sp^2^-character of the C9-atom, bent out of plane by 38.4°. The steric repulsion between the hydrogen atoms at the ortho-position of the phenyl substituent and the hydrogen atoms of the fluorenyl moiety contributes to its rotation. Similar to the **Fl**^**+**^ cation, the absorption bands in the 600–1000 cm^–1^ range are mainly originating from C–H out-of-plane rocking modes. The absorption bands in the 1000–1500 cm^–1^ region originate from the bending and stretching modes of C–H and C–C moieties, while C=C stretches are visible in the 1500–1600 cm^–1^ region. Interestingly, the absorption band at 1508 cm^–1^ originates from the C=C stretch of the bond that connects the fluorenyl and phenyl groups. This C–C bond with a length of 1.43 Å is significantly shorter than regular C–C single bonds (1.54 Å). The high double bond character indicates that the **PhFl**^**+**^ cation is stabilized by the positive mesomeric effect of the phenyl group. This phenomenon has previously been observed in benzylium moieties.^18^ The experimental features around 1580 cm^–1^ are shifted by ∼20 cm^–1^ compared to the computed spectrum at the CAM-B3LYP+D3/def2-TZVPP level of theory of the singlet ion. However, the position of these harmonic frequencies is strongly dependent on the employed level of theory, as showcased in Figure S3.

The **HOFl**^**+**^ cation is planar and *C*_*S*_-symmetric, as the protonation of the precursor is breaking the *C*_2*v*_-symmetry of the fluorenone precursor. Interestingly, its experimental IR spectrum displayed in Figure 2c shares many similarities with the spectrum of **PhFl**^**+**^. As a consequence, the origin of the absorption bands is largely the same except for the absorption bands at 615 and 1538 cm^–1^, which originate from an out-of-plane O–H rocking mode and a C–O stretching mode with partial double bond character, respectively. This C–O bond (1.29 Å) is significantly shorter than C–O single bonds (1.43 Å), highlighting the strong stabilization of the cationic charge at C9 by a positive mesomeric effect.

For the fluorenyl cation and its derivatives, chemical intuition suggests a singlet “dienylic” structure. Such a structure can naively be regarded as two aromatic benzene moieties, connected by a single bond and a bridging carbon atom, which also acts as a charge carrier. Both an “allyl” structure or a triplet electronic state would disrupt conjugation and aromaticity in the two benzene moieties and is, therefore, energetically disfavored. A categorization into aromatic or antiaromatic according to the Hückel rules is compelling but does not do justice to the complexity of this system. As previously reported,^4,19,20^ a classification into nonaromatic is probably most accurate.

In conclusion, bare 9-fluorenyl, 9-phenyl-9-fluorenyl and 9-hydroxy-9-fluorenyl cations were generated and isolated in the gas phase. Their subsequent analysis by cryogenic IR spectroscopy in helium nanodroplets yielded IR spectra containing narrow and highly resolved absorption bands, offering a large amount of structural information. Comparison with DFT calculations shows that in all cases intact ions are probed in their singlet state. The structural characterization of these elusive ions directly shows that the 9-phenyl and 9-hydroxyl-9-fluorenyl cations are substantially stabilized by their substituent in contrast to the 9-fluorenyl cation. This study shows that cryogenic vibrational spectroscopy in combination with mass spectrometric methods is a viable tool to generate and analyze the structure of transient species.

## Experimental Section

The 9-fluorenyl cation **Fl**^**+**^, the 9-phenyl-9-fluorenyl cation **PhFl**^**+**^, and the 9-hydroxy-9-fluorenyl cation **HOFl**^**+**^ are generated by positive ion mode nESI followed by in-source fragmentation of 9-fluorenyl methacrylate (Sigma-Aldrich, 97%) and 9-bromo-9-phenylfluorene (Sigma-Aldrich, 97%) and protonation of 9-fluorenone (Sigma-Aldrich, 98%), respectively (Figure S1). The precursors were dissolved in acetonitrile:water (9:1, v:v) to yield 200 μM solutions. After their generation, the ions are mass-to-charge selected by a quadrupole mass filter and injected into a hexapole ion trap that is cooled to 90 K. Helium nanodroplets (∼10^5^ He atoms) are generated by a cryogenic pulsed valve (19 K) and directed through the ion trap where ion pick-up takes place. Due to their high kinetic energy, the ion-doped droplets can escape the longitudinal trapping potential (∼3 V) and travel further downstream where they are overlapped with the IR beam of the FHI-FEL.^21^ When the IR wavelength of the laser is resonant with an IR-active vibrational mode of the ion, absorption of photons can take place, leading to helium evaporation and the release of the ions. Subsequently, these ions are detected in a time-of-flight mass spectrometer with isotope resolution. This process requires the absorption of many photons. Nonetheless, due to the fast relaxation of the energy (<1 ns) and the long FEL macropulse (∼10 μs), each absorption event will occur from a cold (0.4 K) ion in its vibrational ground state and narrow absorption lines are expected. IR spectra are then obtained by plotting the mass-to-charge selected ion signal as a function of the IR wavenumber. Spectra were recorded in the 600–1700 cm^–1^ range.

## Computational Methods

The computational data shown in the main text, including optimized structures and harmonic frequencies of singlet and triplet states of the ions, are obtained using the CAM-B3LYP hybrid exchange–correlation functional,^22^ D3(BJ) dispersion correction^23^ (which is invoked with the “GD3BJ” keyword in Gaussian 16), and def2-TZVPP basis set (Figure S5).^24^ Additionally, the data in the Supporting Information includes geometry optimizations and harmonic frequency calculations performed at the PBE0^25^ and B3LYP^26,27^ levels of theory of **Fl**^**+**^, **PhFl**^**+**^, and **HOFl**^**+**^ (Tables S1 and S2 and Figures S2–S4). For **Fl**^**+**^ and **HOFl**^**+**^, anharmonic frequency calculations are performed using the GVPT2 method^28−30^ (Tables S2 and S4 and Figures S2 and S4). All calculations are performed using Gaussian 16,^31^ and geometry optimizations are done using tight convergence criteria. Vibrational frequencies are scaled by an empirical factor of 0.965.

## Supporting Information Available

Extended mass spectra, computed energetics and harmonic frequency positions, spectra, and structures (PDF)
Atomic coordinates file (XYZ)
Transparent Peer Review report available (PDF)

## Supplementary Material

jz3c02928_si_001.pdf

jz3c02928_si_002.xyz
