# Supplementary material for: Infrared Spectroscopy of Fluorenyl Cations at Cryogenic Temperatures
Source: J Phys Chem Lett. 2023 Dec 8;14(50):11313–7. doi: 10.1021/acs.jpclett.3c02928 (PMC10749476; doi:10.1021/acs.jpclett.3c02928)
Supplement: Supplementary file 1 — jz3c02928_si_001.pdf [file jz3c02928_si_001.pdf]

Supplementary Information

Infrared Spectroscopy of Fluorenyl Cations at Cryogenic Temperatures

Kim Greis,*[a],[b],† Carla Kirschbaum,[a],[b],‡ Katja Ober,[a] Martín I. Taccone,[a] América Y. Torres-Boy,[a] Gerard Meijer,[a] Kevin Pagel,[a],[b] and Gert von Helden*[a]

(a) Fritz-Haber-Institut der Max-Planck-Gesellschaft, Faradayweg 4-6, 14195 Berlin, Germany
(b) Institut für Chemie und Biochemie, Freie Universität Berlin, Altensteinstraße 23a, 14195 Berlin, Germany

Current addresses:

† Laboratory of Organic Chemistry, Department of Chemistry and Applied Biosciences, ETH Zürich, Vladimir-Prelog-Weg 10, 8093 Zürich, Switzerland

‡ Kavli Institute for Nanoscience Discovery, University of Oxford, South Parks Rd, Oxford OX1 3QU, United Kingdom

Correspondence to: helden@fhi-berlin.mpg.de and greiskim@fhi-berlin.mpg.de

Mass Spectra

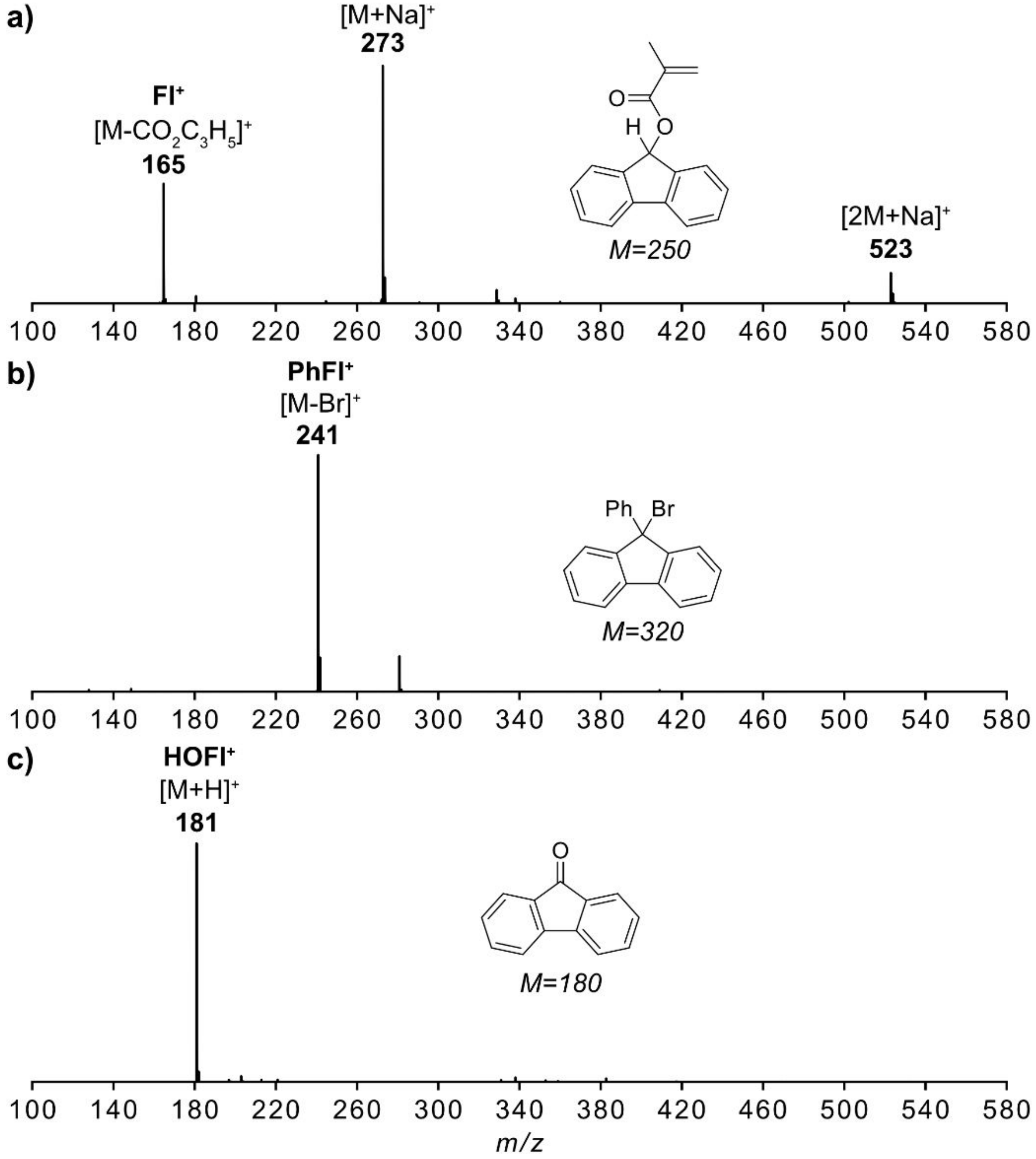

Figure S1. Electrospray ionization (+) mass spectra of (a) 9-fluorenyl methacrylate, (b) 9-bromo-9-phenylfluorene, and (c) 9-fluorenone.

Infrared Spectra and Computed Data

When "+D3" is indicated, we used the Gaussian keyword "EmpiricalDispersion=GD3BJ".

Table S1 Relative energies of the singlet and triplet states of the **Fl⁺**, **PhFl⁺**, and **HOFl⁺** cations in kJ mol^{-1}. The energies are indicated as single-point energies of optimized structures at the respective level of theory. All computations were carried out using the GD3BJ dispersion correction and the Def2-TZVPP basis set. The absolute zero-point vibrational energy (ZPVE) computed at CAM-B3LYP is shown as well.

Ion	Method	Singlet (dienyl)	Triplet
**Fl$^+$**	PBE0	0.0	+72.3
	B3LYP	0.0	+75.3
	CAM-B3LYP	0.0	+77.4
	ZPVE	469.5	465.3
**PhFl$^+$**	PBE0	0.0	+99.1
	B3LYP	0.0	+101.1
	CAM-B3LYP	0.0	+103.3
	ZPVE	686.8	682.2
**HOFl$^+$**	PBE0	0.0	+126.6
	B3LYP	0.0	+125.8
	CAM-B3LYP	0.0	+133.7
	ZPVE	484.4	478.4

Table S2 Position of the experimental infrared absorption bands for the 9-fluorenyl cation **Fl⁺** (C_{2v} symmetry) and comparison to data from matrix isolation spectroscopy and computed frequencies of the singlet state. Only the bands in the experimentally probed region (600–1700 cm^{-1}) are indicated. All frequencies are given in cm^{-1} and computed intensities (*I*) in km mol^{-1}. Computed absorptions are indicated for the singlet state if its intensity is higher than 10 km mol^{-1}.

$\tilde{\nu}$(Exp.)	$\tilde{\nu}$(LDA ice)[a]	$\tilde{\nu}$(PBE0) (*I*)[b]	$\tilde{\nu}$(B3LYP) (*I*)[c]	$\tilde{\nu}$(CAM-B3LYP) (*I*)[d]	$\tilde{\nu}$(GVPT2) (*I*)[e]	Symmetry
700	–	703 (55)	702 (54)	714 (55)	688 (32)	B1
770	–	770 (59)	769 (58)	781 (63)	786 (77)	B1
985	986	984 (63)	979 (70)	991 (99)	1013 (55)	B2
–	–	988 (10)	–	–	–	B1
1009	–	–	1003 (16)	1009 (70)	1031 (57)	B2
1072	1077	1072 (230)	1067 (210)	1065 (404)	1088 (199)	B2
1115	1117	1117 (48)	1113 (55)	1112 (14)	–	B2
1166	1164	1153 (68)	1155 (77)	1160 (115)	1187 (58)	B2
1239	1235	1234 (84)	1223 (67)	1233 (122)	1254 (67)	B2
1267	–	1284 (17)	1270 (24)	1262 (60)	1285 (22)	B2
–	–	1286 (16)	1265 (13)	1279 (11)	–	A1
–	–	–	–	1294 (10)	1321 (11)	A1
1343	1344	1330 (141)	1327 (98)	1336 (203)	1363 (161)	B2
–	–	1399 (11)	1386 (13)	1400 (18)	1424 (22)	A1
–	–	1428 (10)	1424 (18)	–	–	B2
–	–	1446 (10)	–	–	–	A1
1473	1469	1468 (76)	1460 (55)	1480 (105)	1501 (55)	B2
1500	1491	1503 (92)	1478 (90)	1504 (108)	1521 (53)	A1
1572	1576	1585 (468)	1562 (393)	1591 (618)	1613 (215)	B2
1583	–	1607 (127)	1588 (161)	1621 (99)	1641 (80)	B2
1599	1601	1618 (37)	1593 (35)	1632 (42)	1652 (32)	A1

[a] Taken from ref. [1] (no spectral features below 986 cm^{-1} indicated). Computed at [b] PBE0+D3/def2-TZVPP (scaling factor: 0.965), [c] B3LYP+D3/def2-TZVPP (scaling factor: 0.965), [d] CAM-B3LYP+D3/def2-TZVPP (scaling factor: 0.965), and [e] CAM-B3LYP+D3/def2-TZVPP using the GVPT2 method (unscaled anharmonic fundamental frequencies).

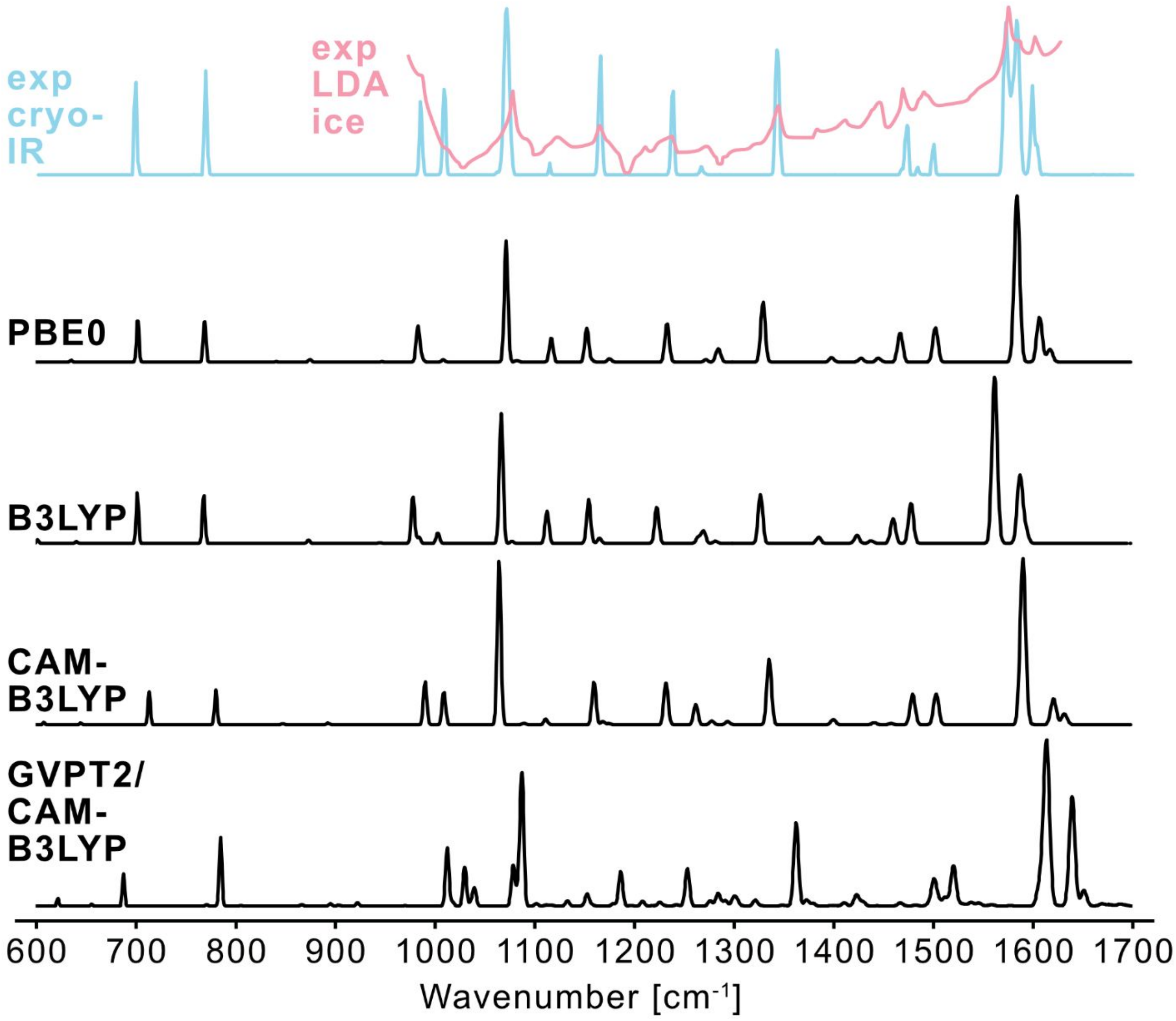

Figure S2. The experimental cryogenic infrared spectrum of the 9-fluorenyl cation **Fl⁺** (blue) is compared to an experimental spectrum from matrix isolation spectroscopy (red) and computed scaled harmonic frequencies of the singlet state at the PBE0+D3/def2-TZVPP, B3LYP+D3/def2-TZVPP, and CAM-B3LYP+D3/def2-TZVPP levels of theory and unscaled anharmonic frequencies at CAM-B3LYP+D3/def2-TZVPP using the GVPT2 method. Except for the vibrations below 800 cm^{-1}, anharmonic frequencies are consistently blueshifted by ca. 20 cm^{-1}, compared to the experimental and scaled harmonic traces. The experimental spectrum recorded using matrix isolation spectroscopy in LDA ice has been adapted from Costa et al.[1]

Table S3 Position of the experimental infrared absorption bands for the 9-phenyl-9-fluorenyl cation **PhFl⁺** (C_2 symmetry) and comparison to the position of scaled harmonic frequencies of the singlet state. Only the bands in the experimentally probed region (600–1700 cm^{-1}) are indicated. All frequencies are given in cm^{-1} and computed intensities (I) in km mol^{-1}. Computed absorptions are indicated for the singlet state if its intensity is higher than 10 km mol^{-1}.

$\tilde{\nu}$(Exp.)	$\tilde{\nu}$(CAM-B3LYP) (I)[a]	Symmetry
623	625 (29)	A
690	698 (32)	A
727	739 (121)	A
–	776 (14)	A
814	831 (19)	A
845	857 (24)	A
993	995 (24)	A
1004	1003 (35)	A
1090	1084 (226)	A
1177	1172 (49)	A
1190	1186 (20)	A
1216	1207 (183)	A
1260	1251 (59)	A
–	1285 (11)	
1290	1290 (19)	
1322	1310 (62)	A
1333	1332 (26)	
1387	1386 (509)	A
1399	1391 (70)	A
–	1454 (24)	A
1450	1456 (37)	A
1467	1472 (70)	A
1507	1510 (534)	A
–	1576 (14)	
1577	1594 (215)	A
1586	1598 (120)	A
1597	1608 (184)	A
–	1620 (118)	A

[a] Computed at CAM-B3LYP+D3/def2-TZVPP (scaling factor: 0.965).

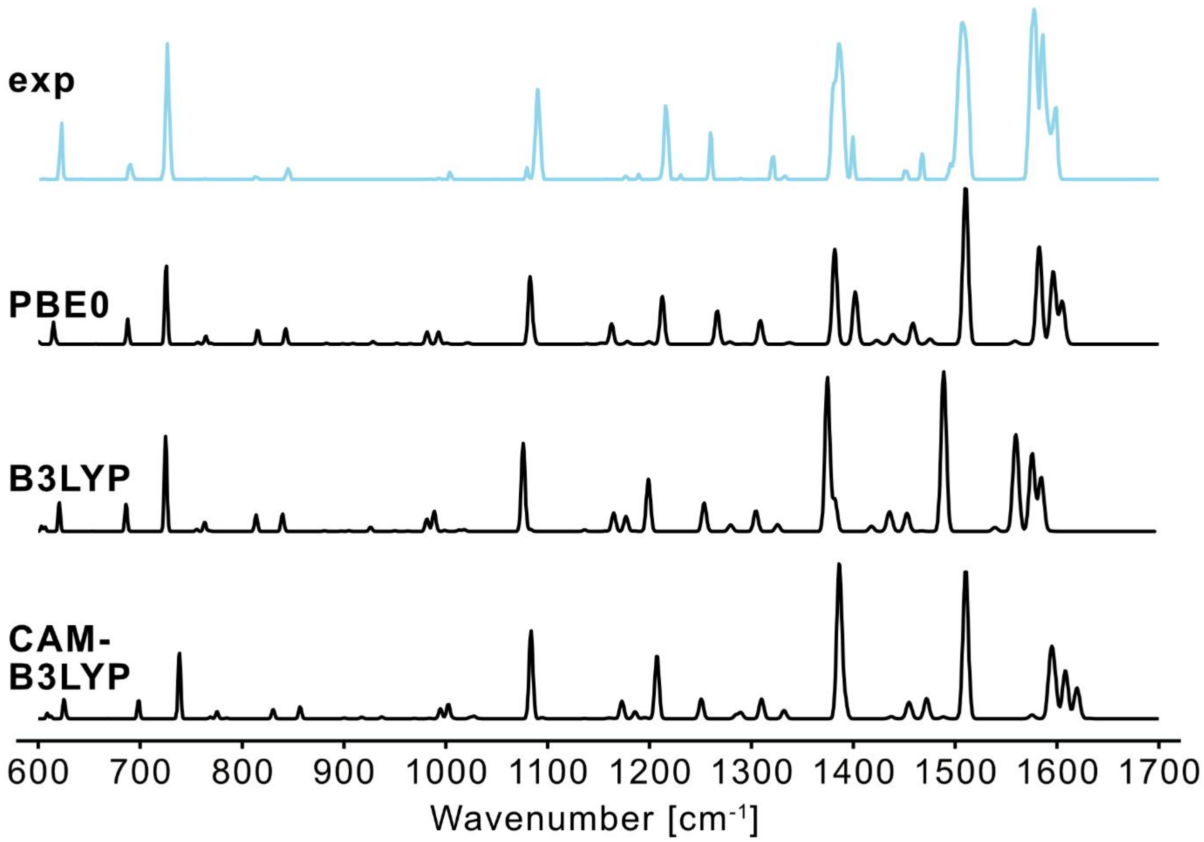

Figure S3. The experimental cryogenic infrared spectrum of the 9-phenyl-9-fluorenyl cation **PhFl⁺** is compared to computed scaled harmonic frequencies of the singlet state at the PBE0+D3/def2-TZVPP, B3LYP+D3/def2-TZVPP, and CAM-B3LYP+D3/def2-TZVPP levels of theory.

Table S4 Position of the experimental infrared absorption bands for the 9-hydroxy-9-fluorenyl cation **HOFl⁺** (C_S symmetry) and comparison to the position of computed frequencies of the singlet state. Only the bands in the experimentally probed region (600–1700 cm^{-1}) are indicated. All frequencies are given in cm^{-1} and computed intensities (*I*) in km mol^{-1}. Computed absorptions are indicated for the singlet state if its intensity is higher than 10 km mol^{-1}.

$\tilde{\nu}$(Exp.)	$\tilde{\nu}$(CAM-B3LYP) (*I*)[a]	$\tilde{\nu}$(GVPT2) (*I*)[b]	Symmetry
615	619 (78)	620 (94)	A″
–	625 (17)	642 (16)	A′
725	738 (130)	751 (119)	A″
–	814 (16)	–	A″
900	897 (52)	918 (49)	A′
1000	1001 (17)	1026 (14)	A′
–	1082 (41)	1107 (35)	A′
–	1139 (18)	1162 (33)	A′
–	1168 (55)	–	A′
1171	1171 (88)	1187 (102)	A′
1218	–	1190 (360)	–
1255	1247 (35)	1267 (27)	A′
–	1283 (35)	1300 (27)	A′
1365	1350 (303)	1366 (85)	A′
–	1448 (29)	1468 (12)	A′
–	1455 (35)	1477 (29)	A′
–	1476 (35)	1500 (12)	A′
1479	1483 (99)	1507 (81)	A′
1538	1541 (555)	1562 (414)	A′
–	1609 (43)	1632 (50)	A′
1602	1613 (316)	1634 (300)	A
–	1622 (70)	1644 (85)	A′

[a] Computed at CAM-B3LYP+D3/def2-TZVPP (scaling factor: 0.965) and [b] CAM-B3LYP+D3/def2-TZVPP using the GVPT2 method (unscaled anharmonic fundamental frequencies).

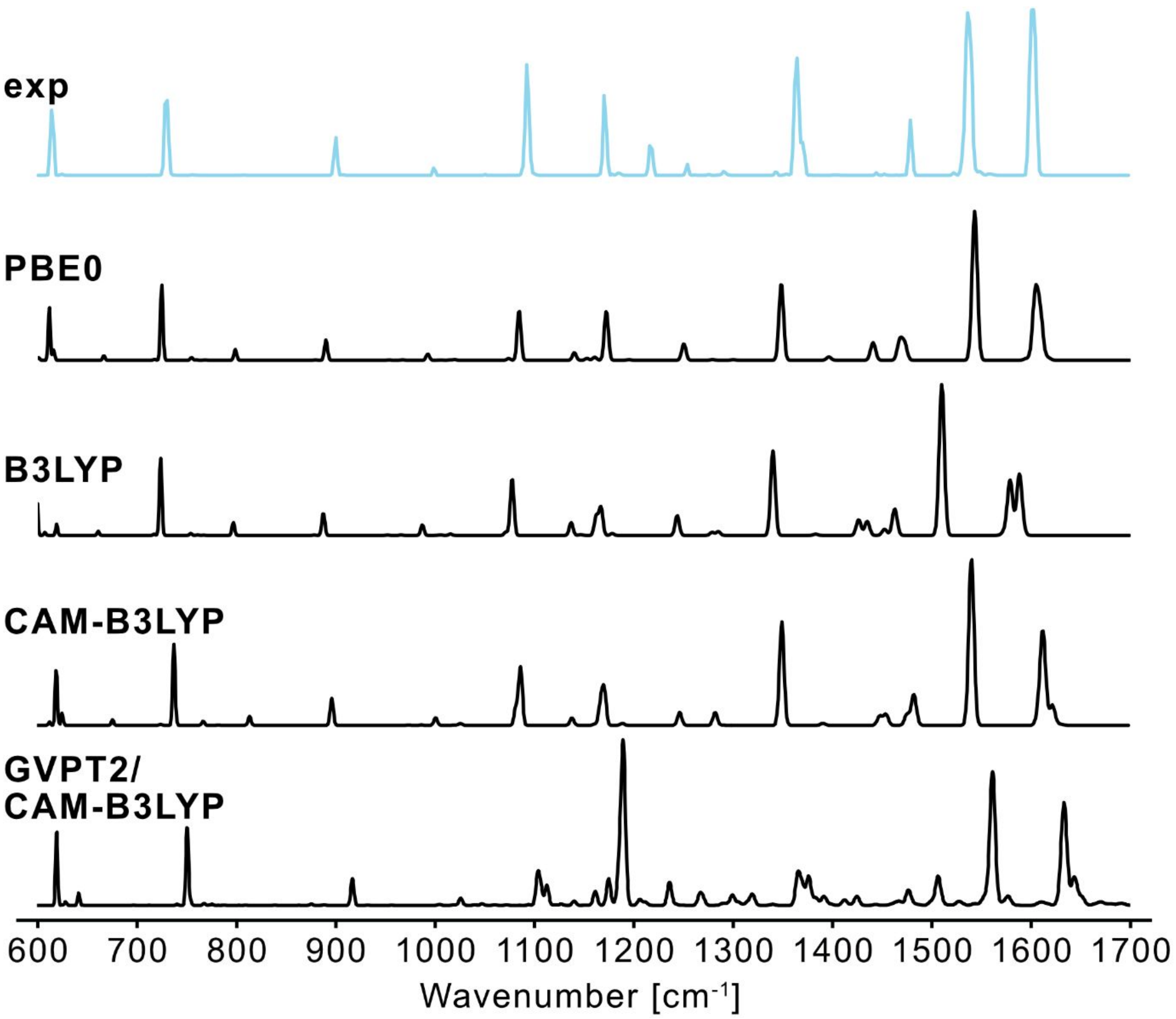

Figure S4. The experimental cryogenic infrared spectrum of the 9-hydroxy-9-fluorenyl cation **HOFl⁺** is compared to computed scaled harmonic frequencies of the singlet state at the PBE0+D3/def2-TZVPP, B3LYP+D3/def2-TZVPP, and CAM-B3LYP+D3/def2-TZVPP levels of theory and unscaled anharmonic frequencies at CAM-B3LYP+D3/def2-TZVPP using the GVPT2 method. Except for the vibrations below 800 cm^{-1}, the anharmonic frequencies are consistently blueshifted by ca. 20 cm^{-1}, compared to the experimental and scaled harmonic traces.

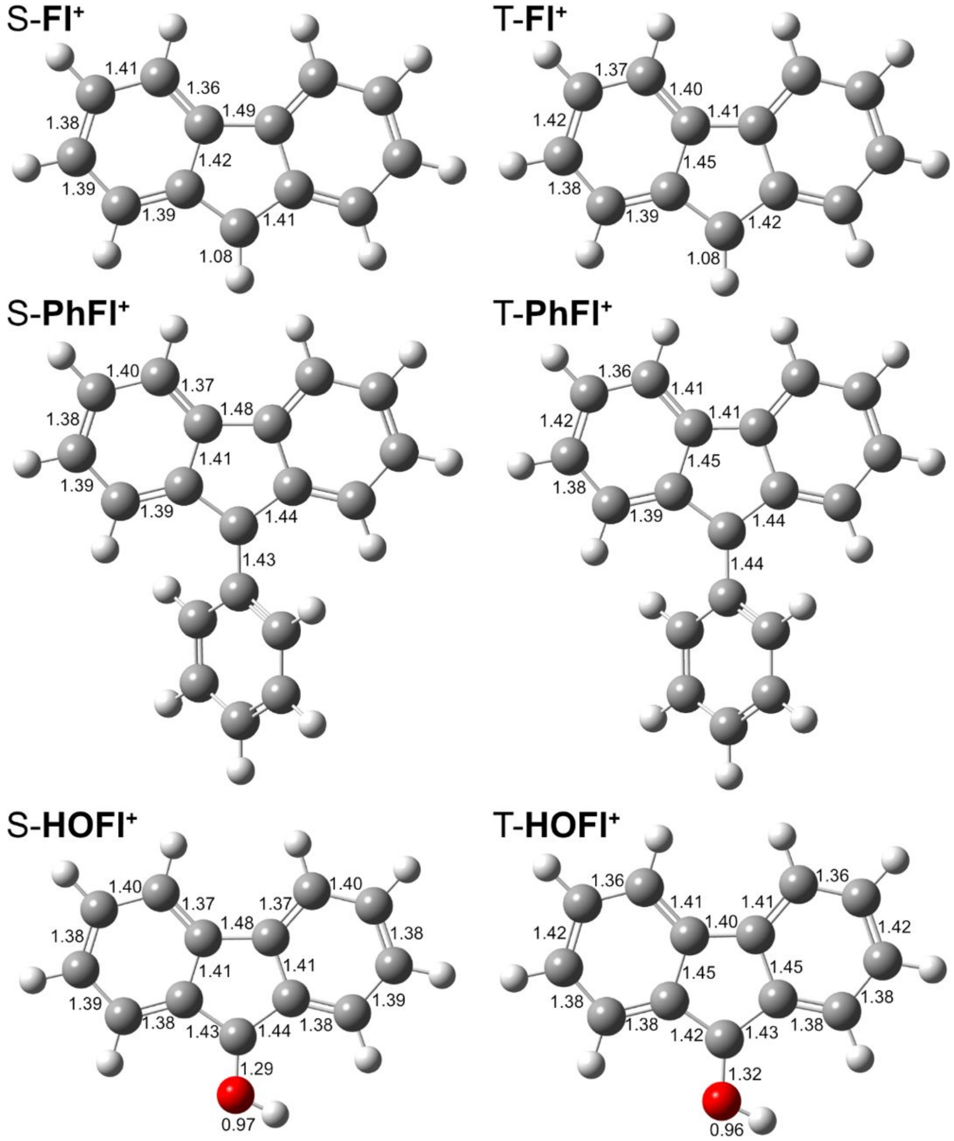

Figure S5. Optimized geometries of the singlet (S) and triplet (T) states of the 9-fluorenyl $\mathbf{Fl}^{+}$ (C_{2V} symmetry), the 9-phenyl-9-fluorenyl $\mathbf{PhFl}^{+}$ (C_2 symmetry), and the 9-hydroxy-9-fluorenyl cation $\mathbf{HOFl}^{+}$ (C_S symmetry) at the CAM-B3LYP+D3/def2-TZVPP level of theory. Bond lengths are indicated in Å.

xyz-Coordinates of Optimized Structures

The xyz-coordinates of $\mathbf{Fl}^{+}$, $\mathbf{PhFl}^{+}$, and $\mathbf{HOFl}^{+}$ cations in their singlet and triplet states optimized at the CAM-B3LYP+D3/def2-TZVPP level of theory can be found in the separate supporting information document "coordinates.xyz".

References

[1] P. Costa, I. Trosien, M. Fernandez-Oliva, E. Sanchez-Garcia, W. Sander, *Angew. Chem. Int. Ed.* **2015**, *54*, 2656-2660.
