# Supplementary material for: Infrared Spectroscopy of Fluorenyl Cations at Cryogenic Temperatures
Source: J Phys Chem Lett. 2023 Dec 8;14(50):11313–7. doi: 10.1021/acs.jpclett.3c02928 (PMC10749476; doi:10.1021/acs.jpclett.3c02928)
Supplement: Supplementary file 3 — jz3c02928_si_003.pdf [file jz3c02928_si_003.pdf]

jz-2023-02928u.R1

Name: Peer Review Information for "Infrared Spectroscopy of Fluorenyl Cations at Cryogenic Temperatures"

First Round of Reviewer Comments

Reviewer: 1

Comments to the Author

In this manuscript, the authors report the spectroscopy of three fluorenyl cations (Fl+). Comparison with theory demonstrates that the ions are all in their singlet spin states. The authors also address whether these species are aromatic or antiaromatic. The work is presented well and can be published after the authors consider suggestions for making their paper a bit clearer.

1) I had to look up pKR, so I would suggest they mention this is the pKa of the side chain.

2) Page 1, second column, the authors state “These studies suggest that the fluorenyl cation is rather non-aromatic.” From the descriptions provided, it is unclear to me how these studies have shown that. Please elucidate further.

3) Figure 2. I think the shading on the energies detracts from legibility and the font size here should be bigger. In part b, I would include the phenyl ring on the structure shown as this would better illustrate the steric effects mentioned in the text.

4) Page 3, column 2. “… at 615 at 1538 cm-1,…” Is the second “at” supposed to be “and”?

5) The agreement between theory and experiment is really quite good with the notable exceptions of the bands near 1500 cm-1 for R = H and Ph. The authors really should provide some comment on what is going on here, especially for part a where there is an extra band observed but not predicted.

6) At the end of the main paper, I am left hanging. Do the authors conclude that these three Fl+ species are aromatic, anti-aromatic, or non-aromatic? This could be stated either at the end of the second to last paragraph or in the “conclusion” paragraph.

7) In the conclusion paragraph, the authors state “The structural characterization of these elusive ions directly shows that the 9-phenyl and the 9hydroxy-9-fluorenyl cations are substantially stabilized by their substituent in contrast to the 9-fluorenyl cation.” Earlier on this page, the authors do clarify why the phenyl substituent provides stabilization, but the paragraph for the OH substituent does not provide any insight into the conclusion made.

8) Computational methods: If the authors performed calculations at different levels of theory, please provide us with a quick synopsis of the results compared to the CAM-B3LYP results and why the latter were chosen for comparison in the main text.

9) Computational methods: How was the scaling factor 0f 0.965 chosen?

Reviewer: 2

Comments to the Author

Greis et al. present new IR spectra of the gas-phase fluorenyl cation and two of its derivatives. The spectra are of very high quality and improve very significantly upon spectra of these species reported in the literature, thanks to the unique instrument constructed by von Helden and coworkers: the combination of a tunable IR free electron laser, a tandem mass spectrometer and a He-droplet molecular beam. I believe that this is a beautiful demonstration of the state-of-the-art in gas-phase ion spectroscopy and how it can be applied to questions of fundamental physical chemistry nature: the singlet versus triplet character of these molecular ions that are classified as anti-aromatic in the Hückel definition. As such, I believe that this study deserves publication in JPCL.

My only concern is the slightly naïve tone of the introduction. Clearly, molecular theory has progressed beyond Hückel MO theory, even though its concepts are still widely in use. For instance, it is well known that many poyaromatics, such as pyrene and coronene, that are anti-aromatic in the Hückel sense, are stable molecules with a singlet electronic ground state. In fact, the authors make use of computational methods (DFT) that are well beyond the Hückel approximation and that indeed clearly indicate a singlet ground state for the cationic systems addressed in this study. In that sense, the finding of a singlet ground state as presented here is not so surprising. Nonetheless, the spectroscopic evidence provided in this Letter is very elegant and extends such evidence to anti-aromatic polyaromatics in cationic charge states.

Despite the somewhat naïve introduction, I do like the Hückel-based conceptual rationale for the observed singlet character of the ground state given towards the conclusion of the paper: the fluorenyl cation is regarded as two separate benzene moieties, which are then both aromatic in the Hückel sense. In this regard, the manuscript is nicely tied together.

A few small points that deserve attention:

The Computational section mentions anharmonic frequency calculations that have been performed. Nonetheless, the main paper shows only results from scaled-harmonic calculations. Why do expensive

theory if you do not use it? Also, is the (uniform) scaling factor used here adopted from literature or is this the best fit?

The caption to Fig 2 mentions that the energies and the structures are from results at the CAM-B3LYP... level. I then assume that the computed spectra are also obtained at that level or are they from one of the other calculations mentioned in the Computational Section?

In the Experimental Section, the nozzle temperature and/or approximate He cluster size could be mentioned as a key experimental parameter.

The description of the Fl+ spectrum in the last full paragraph on page 2 appears to skip over the most striking aspect of the spectra in Fig. 2a: the doublet experimental feature near 1600 versus the single band in the theoretical spectrum. Any comment on this discrepancy?

End of page 2: the tilt angle of the Ph plane relative to the Fl plane should be specified. Supposedly it is small, because otherwise it would disrupt the mesomeric pi-donation mentioned a paragraph ago, right?

page 3, second column, line 3 from top: "615 at 1538" should be "615 and 1538"

Author's Response to Peer Review Comments:

Dear Professor:

Thank you very much for forwarding us the reviewers' comments on our manuscript "Infrared Spectroscopy of Fluorenyl Cations at Cryogenic Temperatures" and the opportunity to submit a revised version.

First, we would like to thank the reviewers for their great effort with the evaluation of this manuscript. Their comments helped to significantly improve the manuscript. The revised manuscript is submitted as a Word file with changes highlighted in yellow. We address the reviewers' requests in the point-by-point response attached to this letter.

Thank you for considering our manuscript for publication in *The Journal of Physical Chemistry Letters*.

With kind regards,

Gert von Helden   Kim Greis

Editorial Requests:

1) Author Affiliations: Please include postal codes/country in the author affiliations in the publication file(s).

#The postal codes and countries have been included for all authors. Furthermore, updated addresses of two of the authors have been provided.#

2) Author Affiliations: Please provide all affiliations in English.

#All affiliations have been provided in English. The name *Freie Universität Berlin* is an official name that does not have an official counterpart in English.#

3) References: In both the main file and the supporting information, fix the style of all references to use JPCL formatting (check all references carefully). ***JPC Letters reference formatting requires that journal references should contain: () around numbers; author names; article title (titles entirely in title case or entirely in lower case); abbreviated journal title (italicized); year (bolded); volume (italicized); and pages (first-last). Book references should contain author names; book title (in the same pattern); publisher; city; and year. Websites must include date of access.

#We adapted the references according to the editor's suggestions.#

4) Supporting Information: Please number SI pages in the following format: "S1, S2…"

#The SI page numbers were adapted as suggested.#

5) TOC Graphic: Please resize the TOC graphic per journal guidelines (2 in x 2 in) and move to the correct position (on the same page as the abstract).

#The TOC graphic was resized as suggested and moved to the same page as the abstract.#

Reviewer 1:

In this manuscript, the authors report the spectroscopy of three fluorenyl cations (Fl+). Comparison with theory demonstrates that the ions are all in their singlet spin states. The authors also address whether these species are aromatic or antiaromatic. The work is presented well and can be published after the authors consider suggestions for making their paper a bit clearer.

#We thank the reviewer for their very positive assessment of our manuscript!#

1) I had to look up pKR, so I would suggest they mention this is the pKa of the side chain.

#We agree with the reviewer that the denotation pKR is not very clear. Based on the cited reference, we decided to modify the sentence more descriptively to "solvolysis rates of the hydroxylated precursors" instead of using abbreviations.#

2) Page 1, second column, the authors state "These studies suggest that the fluorenyl cation is rather non-aromatic." From the descriptions provided, it is unclear to me how these studies have shown that. Please elucidate further.

#We changed the sentence to "The reported NMR-shifts, as well as computed nucleusindependent chemical shift values do not support antiaromaticity in the case of fluorenyl cation derivatives" to indicate based on which observations this conclusion is drawn.#

3) Figure 2. I think the shading on the energies detracts from legibility and the font size here should be bigger. In part b, I would include the phenyl ring on the structure shown as this would better illustrate the steric effects mentioned in the text.

#As suggested by the reviewer we generally increased the font sizes in figure 2 and removed the shading on the energies. Additionally, we included the phenyl ring including its tilted angle in the structure in Figure 2b.#

4) Page 3, column 2. "… at 615 at 1538 cm-1,…" Is the second "at" supposed to be "and"?

#We thank the reviewer for spotting this typo!#

5) The agreement between theory and experiment is really quite good with the notable exceptions of the bands near 1500 cm-1 for R = H and Ph. The authors really should provide some comment on what is going on here, especially for part a where there is an extra band observed but not predicted.

#We agree with the reviewer that it is worthwhile mentioning the discrepancy between experiment and theory in Figures 2a and 2b.

For the pure fluorenyl cation in Figure 2a there are two intense absorption bands in the experiment at 1572 and 1583 cm^{-1}. In the computed spectrum at the CAM-B3LYP level of theory, there is mainly one frequency at 1591 cm^{-1} originating from coupled in-plane C=C stretches of the whole system. Calculations at this and other levels of theory shows that none of them can reproduce these two signals accurately. Even the anharmonic frequencies do not reproduce it. However, there are significant differences in the predicted positions of absorption bands at different levels of theory (Figure S2). The reason for this discrepancy is unclear. We added the following sentences to the manuscript "There are two absorption bands at 1572 and 1583 cm^{-1}, whereas computed harmonic spectra at different levels of theory (Figure S2) mainly predict one matching band in this region. The origin of this discrepancy is unclear..".

For then phenylfluorenyl cation in Figure 2b, on the other hand, we believe that the discrepancy due to experiment and theory is mainly due to the employed level of theory. At other levels of theory, such as PBE0, as shown in the new Figure S3, it becomes apparent that the match of the feature around 1600 cm^{-1} is rather good. With the CAM-B3LYP functional the shape of the feature matches quite well, but it is shifted by ca. 20 cm^{-1}. We added the following sentence to the manuscript: "The experimental features around 1580 cm^{-1} are shifted by ca. 20 cm^{-1} compared to the computed spectrum at the CAM-B3LYP+D3/Def2-TZVPP level of theory of the singlet ion. However, the position of these harmonic frequencies is strongly dependent on the employed level of theory, as showcased in Figure S3."#

6) At the end of the main paper, I am left hanging. Do the authors conclude that these three Fl+ species are aromatic, anti-aromatic, or non-aromatic? This could be stated either at the end of the second to last paragraph or in the "conclusion" paragraph.

#In the second last paragraph, we describe that a triplet state or an allylic structure would disrupt the aromaticity of the annelated benzyl rings. To this paragraph we added the following statement: "A categorization into aromatic or antiaromatic according to the Hückel rules is compelling but does not do justice to the complexity of this system. As previously reported, a classification into non-aromatic is probably most accurate."#

7) In the conclusion paragraph, the authors state "The structural characterization of these elusive ions directly shows that the 9-phenyl and the 9hydroxy-9-fluorenyl cations are substantially stabilized by their substituent in contrast to the 9-fluorenyl cation." Earlier on this page, the authors do clarify why the phenyl substituent provides stabilization, but the paragraph for the OH substituent does not provide any insight into the conclusion made.

#We now include such an explanation for the HOFI+ cation as well: "This C–O bond (1.29 Å) is significantly shorter than C–O single bonds (1.43 Å), highlighting the strong stabilization of the cationic charge at C9 by a positive mesomeric effect."#

8) Computational methods: If the authors performed calculations at different levels of theory, please provide us with a quick synopsis of the results compared to the CAM-B3LYP results and why the latter were chosen for comparison in the main text.

#We now include computed harmonic frequencies at the B3LYP and the PBE0 levels of theory in the Figures S3 and S4. Additionally, we briefly described our decision to mainly show the results using CAM-B3LYP: "For this endeavor, three density functionals are chosen: CAMB3LYP, B3LYP, and PBE0 with the basis set Def2-TZVPP and Grimme's D3 dispersion correction with Becke-Johnson damping. CAM-B3LYP produced overall the best matching harmonic frequencies, whereas some harmonic frequencies are matching better for PBE0 or B3LYP, as shown in Figures S2-S4."#

9) Computational methods: How was the scaling factor 0f 0.965 chosen?

#Empirical scaling factors are necessary to match the harmonic frequencies to the experimentally obtained spectra. The uniform scaling factor of 0.965 has been frequently used for several publications from our lab in the last five years. When used, the harmonic spectra often match well with the experimentally obtained spectra, for several density functionals and basis sets.#

Reviewer 2:

Greis et al. present new IR spectra of the gas-phase fluorenyl cation and two of its derivatives. The spectra are of very high quality and improve very significantly upon spectra of these species reported in the literature, thanks to the unique instrument constructed by von Helden and coworkers: the combination of a tunable IR free electron laser, a tandem mass spectrometer and a He-droplet molecular beam. I believe that this is a beautiful demonstration of the state-of-the-art in gas-phase ion spectroscopy and how it can be applied to questions of fundamental physical chemistry nature: the singlet versus triplet character of these molecular ions that are classified as anti-aromatic in the Hückel definition. As such, I believe that this study deserves publication in JPCL.

#We thank the reviewer for the very positive assessment of our work!#

My only concern is the slightly naïve tone of the introduction. Clearly, molecular theory has progressed beyond Hückel MO theory, even though its concepts are still widely in use. For instance, it is well known that many poyaromatics, such as pyrene and coronene, that are antiaromatic in the Hückel sense, are stable molecules with a singlet electronic ground state. In fact, the authors make use of computational methods (DFT) that are well beyond the Hückel approximation and that indeed clearly indicate a singlet ground state for the cationic systems addressed in this study. In that sense, the finding of a singlet ground state as presented here is not so surprising. Nonetheless, the spectroscopic evidence provided in this Letter is very elegant and extends such evidence to anti-aromatic polyaromatics in cationic charge states.

#We agree with the reviewer that the Hückel MO theory is an old concept. Nevertheless, it is still used by chemists around the world to explain the stability of certain compounds. As such, we think it is worthwhile using this concept. Additionally, our results, to a certain extent, show that formally antiaromatic compounds can still be readily generated and probed.#

Despite the somewhat naïve introduction, I do like the Hückel-based conceptual rationale for the observed singlet character of the ground state given towards the conclusion of the paper: the fluorenyl cation is regarded as two separate benzene moieties, which are then both aromatic in the Hückel sense. In this regard, the manuscript is nicely tied together.

#We thank the reviewer for their positive comment.# A

few small points that deserve attention:

The Computational section mentions anharmonic frequency calculations that have been performed. Nonetheless, the main paper shows only results from scaled-harmonic calculations. Why do expensive theory if you do not use it? Also, is the (uniform) scaling factor used here adopted from literature or is this the best fit?

#For this small system anharmonic frequency calculations are possible at a reasonable computational cost. Interestingly, the scaled harmonic frequencies are matching the experimental spectra better in terms of positions of the bands than then unscaled anharmonic frequencies. Except for a rather consistent shift of ca. 20 cm^{-1} compared to the scaled harmonic frequencies, the anharmonic frequencies do not reveal any significant additional information. Hence, we can conclude that the harmonic approximation is sufficient to describe fluorenyl cations. The scaling factor has already been discussed for Reviewer 1.#

The caption to Fig 2 mentions that the energies and the structures are from results at the CAM-B3LYP... level. I then assume that the computed spectra are also obtained at that level or are they from one of the other calculations mentioned in the Computational Section?

#All the data that is shown in the manuscript is using data from the CAM-B3LYP calculations. We clarified this in the new version of the manuscript.#

In the Experimental Section, the nozzle temperature and/or approximate He cluster size could be mentioned as a key experimental parameter.

#As suggested by the reviewer, we now mention the nozzle temperature and the approximate He cluster size in the manuscript.#

The description of the Fl+ spectrum in the last full paragraph on page 2 appears to skip over the most striking aspect of the spectra in Fig. 2a: the doublet experimental feature near 1600 versus the single band in the theoretical spectrum. Any comment on this discrepancy?

#As also suggested by Reviewer 1 and discussed in greater detail below their commend this discrepancy is highlighted in greater detail in the updated manuscript.#

End of page 2: the tilt angle of the Ph plane relative to the Fl plane should be specified. Supposedly it is small, because otherwise it would disrupt the mesomeric pi-donation mentioned a paragraph ago, right?

#The angle is 38.4 degrees and is now mentioned in the text and in Figure 2. Despite this out of plane bend, the C-C bond length between the phenyl and the fluorenyl moiety indicates a strong pi donation.# page 3, second column, line 3 from top: "615 at 1538" should be "615 and 1538"

#We thank the reviewer for spotting this typo!.#
